# Infrared spectral analysis of gastrointestinal neuroendocrine tumors reveals diagnostic biomarkers

**DOI:** 10.1038/s41598-025-08260-3

**Published:** 2025-07-07

**Authors:** Abhay Mishra, Priya Kumari Gorai, Parul Kaushal, Rakesh Kumar, Fredrik Nikolajeff, Saroj Kumar, Neerja Rani

**Affiliations:** 1 Department of Biophysics, All India Institute of Medical Sciences, New Delhi, 110029 India; 2 Department of Anatomy, All India Institute of Medical Sciences, New Delhi, 110029 India; 3 Department of Nuclear Medicine, All India Institute of Medical Sciences, New Delhi, India; 4 Department of Health, Education and Technology, Lulea University of Technology, 97187 Luleå, Sweden

**Keywords:** FTIR, GI NET, Spectral marker, PCA, Lipids, Proteins, Carbohydrates, Nucleic acids, Gastrointestinal cancer, Membrane structure and assembly, Diagnostic markers

## Abstract

Gastrointestinal neuroendocrine tumors (GI NETs) remain diagnostically challenging due to limitations of current methods. This study pioneers the application of Fourier-transform infrared (FTIR) spectroscopy for GI NET detection through plasma lipid profiling. Analyzing 22 patients and 8 controls, we identified specific biomarker ratios (I_3015_/I_2929_ and I_3015_/I_1650_) reflecting tumor-associated oxidative stress and membrane alterations. Multivariate analysis revealed excellent diagnostic accuracy (94–96.1% sensitivity, 100% specificity) superior to conventional biomarkers, with PCA showing clear group separation (96.5% variance). The FTIR approach demonstrated significant advantages: rapid analysis (< 5 min), minimal sample requirements (4 μL), and low cost, addressing critical clinical needs. Spectral changes correlated with known lipid metabolism dysregulation in NETs, particularly increased unsaturated fatty acids (3015 cm^−1^) and altered acyl chain packing (2929 cm^−1^). These findings establish FTIR as a practical, label-free alternative to invasive diagnostics, with potential for both early detection and treatment monitoring. The identified lipid signatures not only provide robust diagnostic markers but also suggest new therapeutic targets for NET management. This cost-effective technology could transform clinical practice by enabling routine screening and personalized treatment strategies. Future studies should validate these results in larger cohorts and explore correlations with tumor grade and treatment response.

## Introduction

Neuroendocrine neoplasms (NENs) are rare, heterogeneous tumors originating from cells of the endocrine and nervous systems, most found in the gastrointestinal (GI) tract, pancreas, and lungs^1^. According to the US SEER database, within gastrointestinal tract, small intestine (with ~ 17.3% of all NENs) is the most common and then rectum (15.9%), colon (10.9%), stomach (6%), pancreas (7%), and appendix (3.4%)^2^. As per 2019 WHO classification, GI-NETs have been classified into three grades based on mitotic rate and Ki-67 proliferation index. Gastrointestinal neuroendocrine tumors (GI NETs) represent ~ 70% of all NETs, with an annual incidence of 1–5 cases per 100,000 population globally^3^. While historically considered rare, recent data show a sixfold increase in diagnosis over the past 30 years due to improved techniques and awareness^4^ and the 5-year survival rate is low with higher tumor grades leading to poor prognosis.

The clinical features of GI NETs vary according to their anatomical location and cell type^5^. They are usually accidently detected during surgeries for appendicitis, GI bleeding or perforation^6^. As they present with non-specific symptoms including abdominal pain, diarrhea, fever^7^, making early diagnosis very challenging. General modalities like biomarker assessment, ultrasound, endoscopy, CT, MRIs, radiological and nuclear imaging along with confirmation by histology have aided the diagnosis of GI NETs^8^, but still timely diagnosis of gastrointestinal neuroendocrine tumors (GI NETs) is the most challenging step in planning the treatment of these tumors. Various biochemical tests and molecular markers including Chromogranin A (CgA) that have recently been studied for their potential are being used currently in early GI NETs diagnosis but are ineffective due to their non-specific and sensitive nature^9^. These biomarkers do not predict prognosis and behavior of the tumor, as the heterogeneity of originating cells and secretary capacity of amine and peptides may lead to inaccurate diagnosis of the disease and currently, no one biomarker can accurately confirm the prognosis of GI NETs^10^. Moreover, the biopsy followed by a microscope-based histopathological assessments such as mitotic count and ki67 index are the gold standard for diagnosing GI NETs that may not detect the complex molecular underlying cancer progression. In addition, these procedures are time-consuming and costly. Also relying on histological evaluation based on changes in tissue morphology is not a reliable way to estimate the accurate risk of GI NETs due to susceptibility to inter-observer variability and often identifies GI NETs in its advanced stages^11^. Additionally, the imaging techniques, Ga-68 DOTANOC PET/CT and 18F-FDG-Scan used as confirmatory tests but, these scans are invasive, costly, inaccessible, give exposure to radiation and show difficulty in detecting micro-calcification or small tumors. So, Timely diagnosis of gastrointestinal neuroendocrine tumors (GI NETs) is the most challenging step in planning the treatment of these tumors. Hence, there is need for more advanced investigative techniques diagnosing the GI NETs in early stages and for better management of tumors.

Recent studies have highlighted the scope of metabolic characteristics of bio-macromolecules as diagnostic and prognostic markers for early diagnosis of cancer detection and screening[^12,13^] Study of biochemical changes based on vibrational signatures of their components is being targeted in various biological samples. Additionally, the human body is composed of water, protein, nucleic acid and carbohydrates. Significant research has shown that any changes in these biomolecules could be used as early detection markers before any morphological changes. So, FTIR is better than traditional methods for cancer screening and diagnosis, which could make it a potent clinical tool in modern medicine^14–16^.

Fourier transform infrared spectroscopy (FTIR) is a vibrational technique that utilizes infrared radiation to excite and absorb molecular bonds within a sample. FTIR, particularly in attenuated total reflection (ATR) mode, is a feasible technology for label-free chemical profiling of samples^12^. The FITR is also being exploited for cancer screening as well due to its simplicity, label free nature, non-invasive approach, requiring minimal sample volume. Blood based FITR has been applied to biomedical diagnosis as compared to biopsy samples as it prevents any risk of tumor damage^17^. There is evidence of FTIR based accurate detection of cancer in brain^18^, lung^19^, prostate^20,21^; ovary^22,23^ and breast^24,25^. However, its potential for detecting GI NETs remains unexplored. To our knowledge, no studies on GI NETs have been published in liquid biopsy. This study focuses on examining FTIR spectra of GI NETs blood samples and reporting and comparing results for GI NETs patients and controls to get insight into disease progression.

## Material and methods

### Subject recruitment

**Fig. 1:**
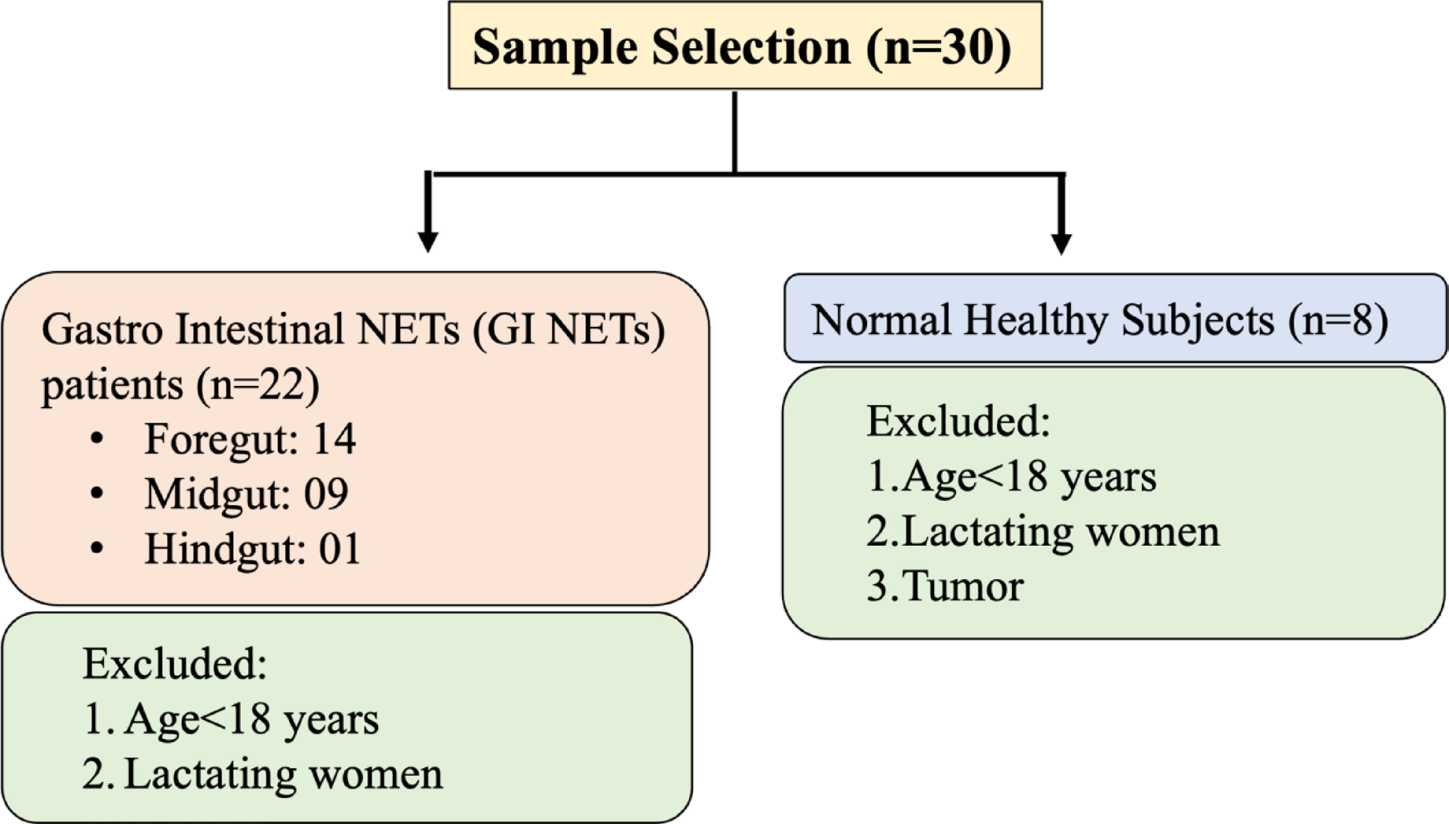
Study enrollment flowchart: This flow chart illustrates the process of subject enrollment, detailing the number of participants screened, those included, and those excluded based on specific inclusion and exclusion criteria. It highlights key stages of participant selection for the study.

A total of 22 patients who were diagnosed with GI NETs at the departments of GI surgery and nuclear medicine, AIIMS Delhi, India and 8 age-matched healthy controls were included in this study. These patients were enrolled within a period of 2 years. The distribution of subjects and exclusion criteria is shown in Fig. 1. Informed consent was obtained from all participants and/or their legal guardians. The study was approved by the Institutional Ethics Committee of All India Institute of Medical Sciences, New Delhi (IEC PG-362/30.08.2018). All methods were carried out in accordance with relevant guidelines and regulations. A written participant information sheet was given to the subjects who agreed to take part in the study by signing on the consent form.

### Sample collection and processing

**Fig. 2:**
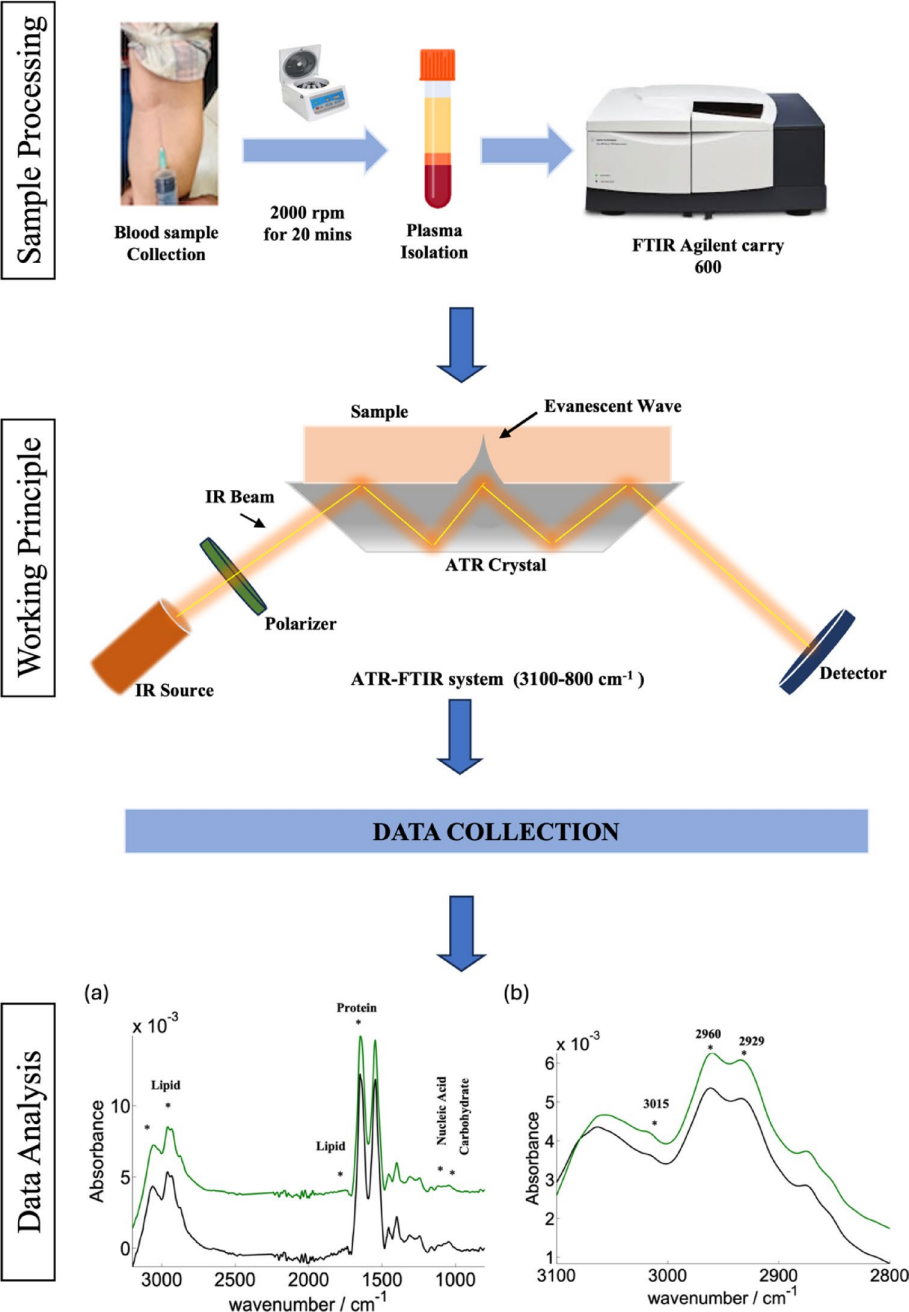
Diagrammatic quick representation of protocol to acquire spectra: This figure presents the graphical depiction of the spectra obtained during the study. It visually represents the key spectral patterns and their corresponding wavelengths or frequencies, showcasing the data collected from the analysis.

5 mL of blood was collected from patients in EDTA vials. The samples were centrifuged at 2000 rpm for 20 min at 4 °C to eliminate the macromolecules. Post centrifugation the supernatant plasma was collected in fresh MCT and stored at − 20 °C for ATR-FTIR (Fig. 2). The grading of patients was done using Ga-68 DOTANOC PET/CT and 18F-FDG-Scan. The Ga-68 DOTANOC PET/CT scan, which is valuable for assessing the presence and extent of disease for staging, was used for categorization of tumor grades based on uptake as follows: intense uptake was classified as Grade I, mild or few uptake sites as Grade II with Ga-68 DOTANOC PET/CT.

### Data acquisition and processing through FTIR

A 4 μL concentrated plasma sample was put on an ATR reflection diamond element. To obtain FTIR spectra, a DTGS detector and an Agilent Cary 600 FTIR apparatus was used with a 4 cm^−1^ resolution. All experiments were performed at room temperature using Agilent Resolution Pro software. The background spectrum for 128 scans of each sample was obtained using a single beam spectrum of the buffer (PBS), in which each sample was resuspended. The instrument was continuously purged with dry air^26^. We studied the spectral characteristics of plasma samples.

The diamond ATR crystal was cleaned with ethanol and then exposed to a background measurement (air was administered, and spectra were acquired as a reference) before a new sample was measured. The sample was left stationary for two minutes, and then measurements were taken at room temperature. We found a nominal resolution of 4 cm^−1^ with spectral wavenumbers ranging from 900 to 3100 cm^−1^. To increase the signal-to-noise ratio, 128 scans were applied to each measurement. Data was processed using GraphPad Prism (v8.0), Agilent Resolution Pro (v5.2), and Erik Goormaghtigh’s Kinetics MATLAB toolbox (v3.1, MathWorks, MA, USA).

The second derivative spectra were generated using Erik Goormaghtigh’s MATLAB software Kinetics, which included a 4 cm^−1^ smoothing function. These spectra were examined in detail to see whether any water vapor features were present, particularly above 1750 cm^−1^. In order to give uniform band amplitudes, the spectra displayed in the photos were normalized^27^.

### Spectral preprocessing method

To enhance spectral integrity, raw FTIR data were preprocessed using established techniques. Savitzky-Golay smoothing (2nd-order polynomial, 5-point window) was applied to reduce high-frequency noise while preserving peak shapes—critical for accurate biomarker identification in complex biological spectra^25^. For baseline correction, a six-point approach was selected after empirical optimization, with anchor points at 3200 cm^−1^, 2700 cm^−1^, 1950 cm^−1^, 1485 cm^−1^, 1000 cm^−1^, and 801 cm^−1^. These wavenumbers were chosen to span key spectral regions (e.g., lipid/protein vibrations, fingerprint zone) while minimizing scattering artifacts. This method ensured consistent baseline flattening without distorting diagnostically relevant peaks.

A detailed visual examination was then performed to identify absorbance bands that revealed significant differences between the experimental groups. This multistep procedure helps to improve data accuracy and dependability in the context of spectral analysis. Height ratios were calculated for each band, and a t-test was used to see if there were any significant differences between groups (p < 0.05). This rigorous preprocessing technique guarantees the reliability and correctness of the following spectrum analysis.

### Statistical analyses

GraphPad Prism 8.0 software was used for all statistical analyses. Unpaired Statistical significance was calculated using the results of the F test and p < 0.05 was considered statistically significant. Multiple parameters can be examined concurrently using the multivariate statistical approach known as Principal Component Analysis (PCA). PCA is widely used in pattern recognition and spectroscopy because it allows for a broader viewpoint than only considering the position and intensity of spectral features. It selects a small number of variables from a big dataset and displays them in a dimensional space of uncorrelated principal components, which are linear combinations of the original variables. Here, metaboAnalyst was utilized for better understanding and interpretation.

A measure of separability is the total area under the receiver operating characteristic curve (ROC), often referred to as area under the curve (AUC). A probability curve called the ROC is used to assess test accuracy. It illustrates how well the test may distinguish between different class levels. The test’s ability to discriminate between people with and without medical issues is enhanced by a greater AUC. Plotting TPR/ True positive results (sensitivity) on the y-axis and FPR/False positive result (specificity) on the x-axis yields the ROC curve. A good test is shown by an AUC that is close to one, indicating a high degree of separability. An AUC that is close to zero suggests a subpar test with the lowest separability score. ROC analyses were employed in this instance to assess the accuracy of different methods.

## Results

### Patient history

**Fig. 3:**
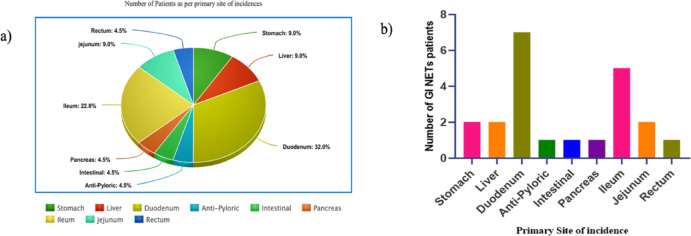
Percentage Distribution of Primary Sites in GEP-NETs: This figure illustrates the distribution of primary sites in gastroenteropancreatic neuroendocrine tumors (GEP-NETs). (**a**) A pie chart displays the percentage distribution of various primary sites, providing a visual overview of the most and least common tumor locations. (**b**) A bar diagram shows the number of GEP-NET patients across different primary sites, offering a comparative view of incidence rates among the patient group.

Subject recruitment, sample collection, and processing for the study were conducted following ethical guidelines. Patients diagnosed with Gastrointestinal Neuroendocrine Tumors (GINET) (n = 22) were selected from the GI surgery and nuclear medicine at AIIMS, India, between 2022 and 2023. The study population comprised an equal distribution of males (n = 11, 50%) and females (n = 11, 50%), with ages ranging from 28 to 94 years, and a mean age of 52.68 ± 3.16 years. A thorough process of informed consent was followed. All participants received a written participant information sheet and provided written consent before inclusion in the study. Clinical characteristics such as tumor grading were determined using DOTANOC PET imaging and Ki-67% values (Table 1, Fig. 3a). Among the 22 GINETs patients, the small Intestine accounts for the most common primary site of incidences with 7 out of 22 cases originating in the duodenum (Fig. 3b).

**Table 1:** Demographic and clinical profile of GINETs patients.

Grades of Patients as per DOTANOC & SUV Max Value			Characteristics	n (%)
Available in 18/22 patients			Total patients	22
Grade I		10/18 (55.5)	Males	11 (50)
Grade II		04/18 (22.2)	Females	11 (50)
Grade III		04/18 (22.2)	Age (in years)	
Number of Patients as per primary site of incidences			Mean	52.68 ± 3.16
Stomach		02/22 (9.09%)	Range	28–94
Liver		02/22 (9.09%)	Anti-Pyloric	01/22 (4.5%)
Duodenum	(D1)	03/07 (42.8)	Intestinal	01/22 (4.5%)
07/22 (31.8%)	(D2)	03/07 (42.8)	Pancreas	01/22 (4.5%)
	(D3)	01/07 (14.2)	Ileum	05/22 (22.7%)
Rectum		01/22 (4.5%)	Jejunum	02/22 (9.09%)

This table presents the demographic characteristics, tumor grading (DOTANOC & SUV Max values), and distribution of primary tumor sites for the 22 patients included in the study.

### Plasma infrared spectra analysis

**Fig. 4:**
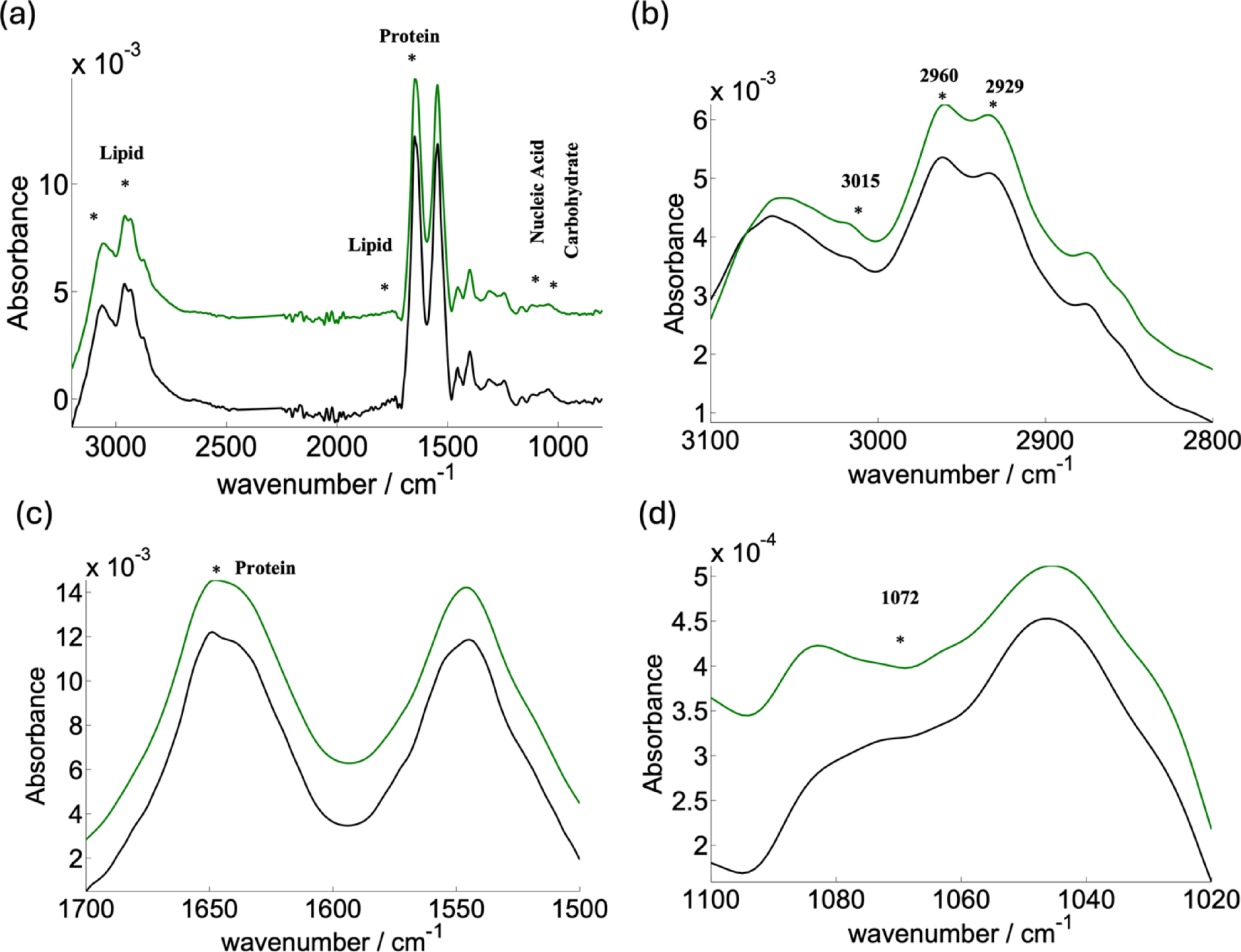
Characteristic spectra with key absorption regions: This figure presents the full spectra and highlights specific regions of interest: (**a**) Full-length spectra from 3100–900 cm^−1^, with a star marking discernible differences. (**b**) Spectra showing lipid absorption (3000–2800 cm^−1^) and regions related to oxidative stress (3015 cm^−1^, 2960 cm^−1^, 2929 cm^−1^). (**c**) Amide I region (1700–1500 cm^−1^) with a prominent peak at 1650 cm^−1^. (**d**) Nucleotide region (1090–1030 cm^−1^) highlighting the absorption at 1072 cm^−1^.

The plasma absorbance spectra for Gastrointestinal Neuroendocrine Tumors (GI NETs) patients and healthy subjects (Control group) are shown in Fig. 4a, represented in green and black, respectively. The spectral band extends from 900 to 3100 cm^−1^, covering a range of molecular vibrations related to lipids, proteins, nucleic acids, and carbohydrates. Notable differences in these regions between the two groups form the foundation for identifying spectral signatures that could aid in early disease detection. The star marker in the figure highlights the regions with the most discernible variations. This approach aligns with previously published studies, particularly those investigating lipid-lipid and lipid-protein interactions using FTIR spectroscopy^12,28^.

#### Baseline corrected spectra for GINETs plasma

FTIR spectroscopy, widely used to study molecular compositions, divides the spectrum into several regions, each corresponding to a specific molecular component. In our analysis, specific regions were linked to key biomolecules such as lipids and proteins, as listed below:

#### Assignment of FTIR band characteristics, GINETs and HCs

Above mentioned bands, especially those in the lipid and protein regions (Fig. 4b, c), provide a window into molecular changes associated with disease states. Such observations corroborate findings from earlier research focused on the implications of lipid and protein structural alterations in pathological conditions.

#### Analysis of lipids changes

The spectra revealed a significant shift in the lipid absorption region between GI NETs patients and the healthy control group, highlighting IR spectroscopy’s potential in identifying lipid profile alterations. Lipid alterations are crucial markers for various diseases, and the ability of IR spectroscopy to analyze multiple lipid components simultaneously allows for a comprehensive assessment. Key lipid regions analyzed in this study include: 3050–3000 cm^−1^: CH olefinic stretch (degree of fatty acid saturation); 3000–2800 cm^−1^ (Fig. 4b): Lipid acyl chain stretching (CH_2_ and CH_3_); 1760–1720 cm^−1^: Carbonyl ester stretching.

**Fig. 5:**
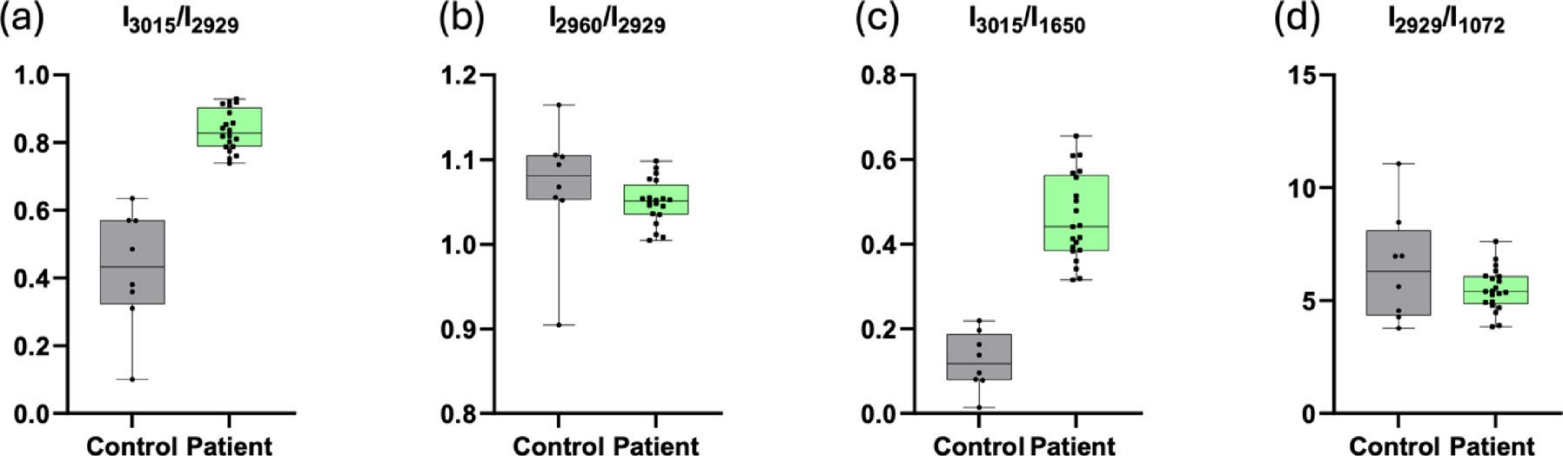
This figure illustrates the relative intensity ratios of specific spectral bands between gastroenteropancreatic neuroendocrine tumors (GINETs) and control subjects: (**a**) Relative intensity ratio I_3015_/I_2929_ (**b**) Relative intensity ratio I_2960_/I_2929_ (**c**) Relative intensity ratio I_3015_/I_1650_ (**d**) Relative intensity ratio I_2929_/I_1072_.

The absorption band at 3015 cm^−1^ is attributed to the = CH vibration (olefinic stretch), a marker of lipid unsaturation, while bands at 2960 cm^−1^ and 2929 cm^−1^ correspond to asymmetric stretches of CH₃ and CH₂ groups, respectively. These vibrational bands provide insights into the structural features of plasma lipids in both healthy and diseased states (21, 22). Using a “spectroscopic lipid-to-lipid ratio,” we computed key ratios such as (I_3015_/I_2929_) (Fig. 5a), representing oxidative stress in relation to lipid acyl chains, and (I_3015_/I_1650_) (Fig. 5c), representing oxidative stress relative to protein content. These ratios allow for a more accurate comparison of lipid changes between groups, eliminating potential artifacts from concentration variations. Key findings: I_3015_/I_2929_ ratio: Higher in GI NETs patients (0.4098 ± 0.04) compared to healthy controls (P < 0.05). I_3015_/I_1650_ ratio: Higher in patients (0.3380 ± 0.04), confirming the presence of oxidative stress and lipid alterations in GI NETs. Moreover, the I_2960_/I_2929_ ratio showed subtle variations (0.01832 ± 0.02), suggesting unique lipidomic fingerprints (Fig. 5b). These data indicate significant changes in lipid composition, with implications for membrane fluidity and function. These alterations are consistent with pathophysiological changes seen in various diseases, including cancer.

Protein analysis revealed subtle variations in their absorption bands in Fig. 5c. In comparison to healthy individuals, a detailed analysis of the total protein composition of the patient’s plasma showed a unique protein profile with a higher absorbance^29^. A different method looks at how amide I affects the asymmetric stretching vibrations of C-H in fatty acids. This analysis suggests that lipid structural changes in plasma also influence membrane protein composition.

The fingerprint region’s spectral information is enhanced by an additional prominent band at 1072 cm^−1^, which provides information on the symmetric phosphate stretching of nucleic acids (Fig. 4d). Interestingly, there were noticeable variations between the GI NETs spectra and the healthy participants in the 1100 cm^−1^ to 1000 cm^−1^ region. When comparing the acyl band (lipid) to the symmetric phosphate (nucleic acid) ratio, it showed the difference was not statistically significant (P < 0.05) (Fig. 5d). These complex spectrum subtleties provide unique molecular markers that illuminate the complex metabolic changes linked to GI NETs.

#### Variation analysis by PCA Plot, Heatmap and ROC curve analysis

To further investigate the discriminative power of the spectral data, we performed Principal Component Analysis (PCA), Heatmap visualization, and Receiver Operating Characteristic (ROC) curve analysis, focusing on specific band ratios (I_3015_/I_2929_, I_3015_/I_1650_) (Table 2). These ratios captured metabolic differences between the two groups with high accuracy.

**Table 2:** Absorption bands and corresponding vibrational modes in spectra.

Assignment	Bands	Vibrational mode
Lipids	3015 cm^−1^	C=C, Olephenic stretch
Lipids	2960 cm^−1^	(CH3 a.s.), Membrane lipids
Lipids	2929 cm^−1^	(CH2 a.s.), Membrane lipids
Protein	1650 cm^−1^	Amide I, Proteins
Nucleic Acids	1072 cm^−1^	phosphate stretching of nucleic acids

This table lists the spectral absorption bands associated with specific vibrational modes, detailing the characteristic frequencies for lipids and proteins as observed in the study.

**Fig. 6:**
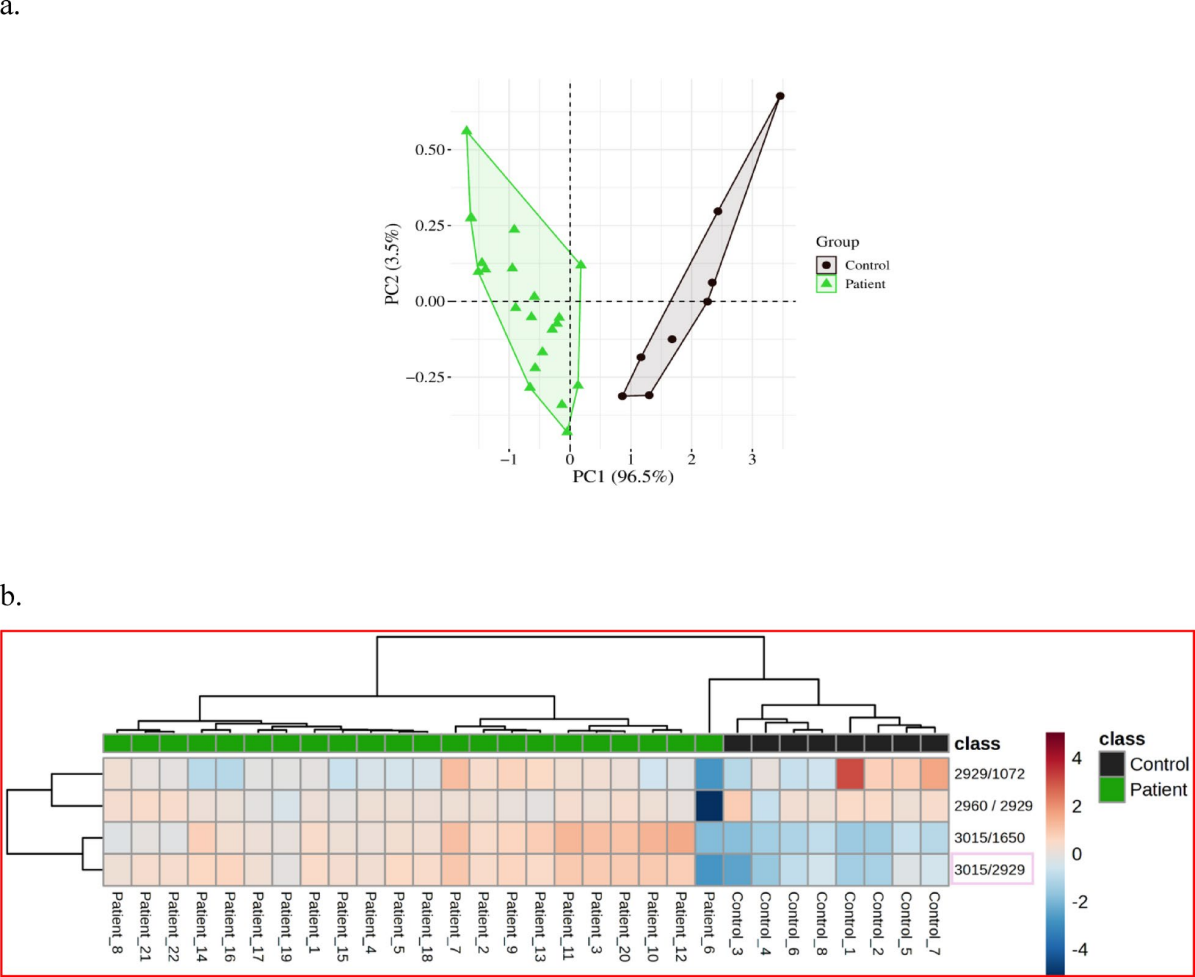

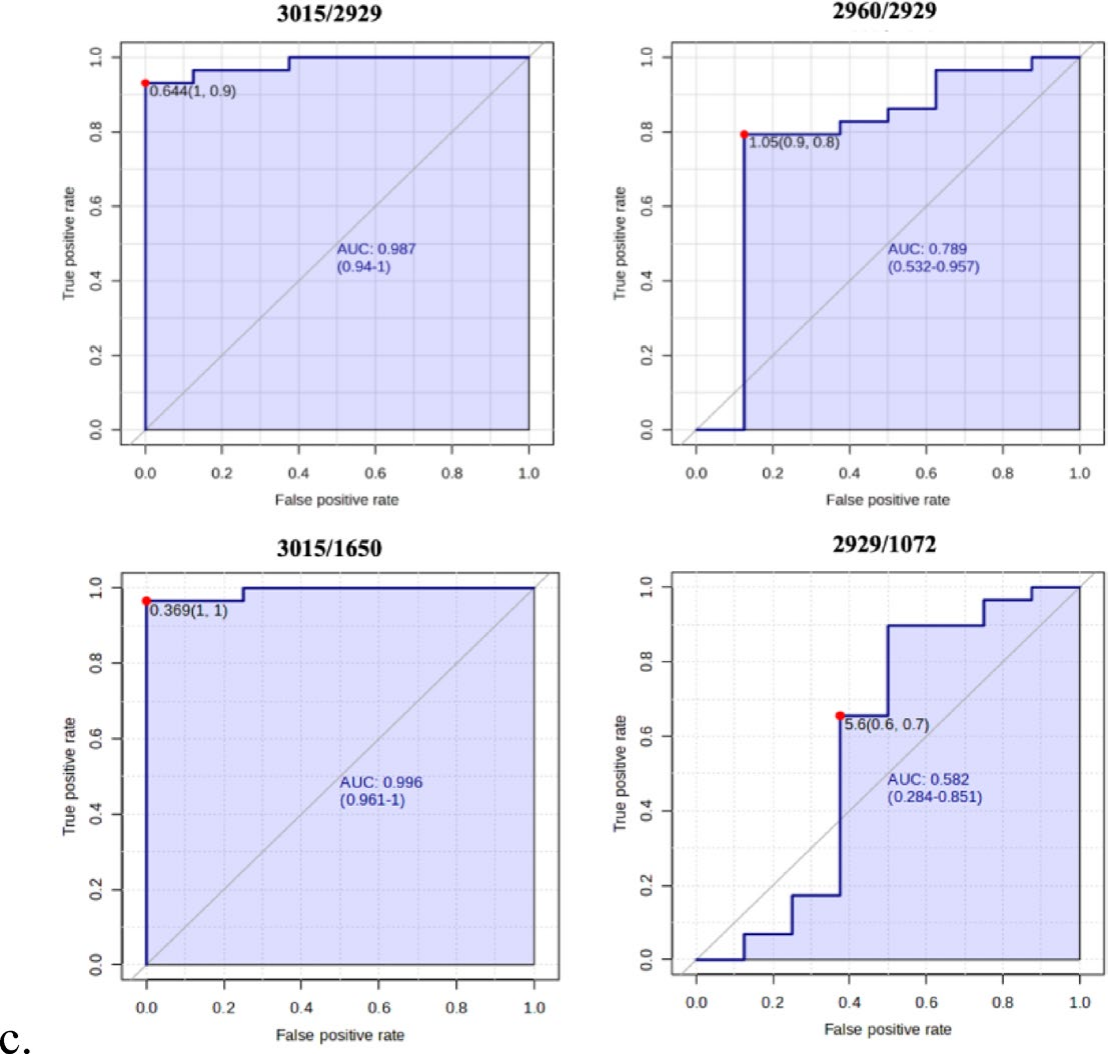
PCA Analysis and Heatmap of Spectral Ratios This figure comprises three components illustrating the relationship between spectral ratios in controls and patients: (**A**) PCA plot analysis comparing spectral ratios for control subjects (n = 8) and patients (n = 22), highlighting the clustering of the two groups based on spectral data. (**B**) A heatmap visualizing the patient data across different clusters, with a color scale representing the percentage of patients and controls for each attribute relative to the corresponding ratios. The scale ranges from the lowest cluster mean (− 2) to the highest cluster mean (2.0), indicating variability within the data. (**C**) The ROC curve illustrating the area under the curve (AUC), specificity, and sensitivity for each corresponding ratio, providing a measure of diagnostic performance.

The PCA plot (Fig. 6a) illustrates the separation between GI NETs patients and healthy controls, with the first component accounting for 96.5% of the variance and the second for 3.5%. The score plot shows clear clustering, with green triangles representing patients and black circles for controls. This strong separation underscores the discriminatory power of the identified spectral ratios. The heatmap (Fig. 6b) visually displays the intensity of spectral band ratios across 22 patients and 8 controls, showing clear distinctions between the two groups. Meanwhile, the ROC curve (Fig. 6c) quantifies the diagnostic accuracy, with the I_3015_/I_2929_ ratio achieving an AUC of 0.987, sensitivity of 94%, and specificity of 100%. Similarly, the I_3015_/I_1650_ ratio showed an AUC of 0.996, sensitivity of 96.1%, and specificity of 100%.

These results validate the utility of FTIR spectral analysis in detecting GI NETs, particularly through lipidomic changes (Table 3). The prominent alterations in lipid profiles offer a promising approach for early disease detection and highlight the role of lipid metabolism in GI NETs pathology. To contextualize the diagnostic performance of FTIR-derived band ratios, we compared our findings with conventional biomarkers such as chromogranin A (CgA). CgA, while widely used, has limitations including variable sensitivity (60–90%) and specificity (60–85%) due to renal disease or drug interference^30^. In contrast, our FTIR-derived I₃₀₁₅/I₂₉₂₉ ratio (sensitivity: 94%; specificity: 100%) avoids these pitfalls by probing structural lipid changes independent of secretion^31^. FTIR’s cost-effectiveness^32^ further supports its translational potential. The superior performance of lipid-derived band ratios (e.g., I_3015_/I_2929_) over CgA may stem from FTIR’s ability to capture global biochemical alterations rather than relying on secretory phenotypes. CgA’s variability in sensitivity (e.g., reduced in proton-pump inhibitor users or low-volume disease) underscores the need for adjunct tools like FTIR, which reflects membrane lipid metabolism independent of hormonal secretion.

**Table 3:** Band ratios and associated biomolecules in GI NETs vs. normal healthy subjects.

Band ratio	Associated biomolecules	Higher in normal healthy	Higher in GI NETs
3015 cm^−1^ / 2929 cm^−1^	(=CH Olefinic) Membrane Lipids/Membrane Lipids acyl chain (CH₂)		✔
2960 cm^−1^/ 2929 cm^−1^	Membrane Lipids (CH₃ a.s.)/Membrane Lipids (CH₂ a.s.)	✔	
3015 cm^−1^/ 1650 cm^−1^	Membrane Lipids (CH₂ a.s.)/Proteins (Amide I)		✔
2929 cm^−1^/ 1072 cm^−1^	Membrane Lipids (CH₂ a.s.)/Nucleic Acids (C–O stretching, C–O bending)	✔	

This table summarizes the band ratios of specific biomolecules, indicating which ratios are higher in patients with gastroenteropancreatic neuroendocrine tumors (GI NETs) compared to healthy controls.

The significant impact of discrete lipid modifications in these results highlights the strong classifier function of the I_3015_/I_2929_ band ratio. It offers more support for the possibility for diagnosis associated with lipidomic changes in plasma.

## Discussion

This study combines Fourier-transform infrared spectroscopy (FTIR) with Principal Component Analysis (PCA) and heatmap visualization to provide preliminary evidence of their diagnostic potential in the early detection of gastrointestinal neuroendocrine tumors (GI NETs). By identifying subtle alterations in the lipid, protein, nucleic acid, and carbohydrate profiles in blood plasma, FTIR has demonstrated the ability to differentiate between GI NET patients and healthy controls. This capability is crucial, as early detection is imperative for timely and effective treatment and management of tumors.

### Lipid alterations and their role in tumor progression

Our findings indicate significant differences in lipid profiles between GI NET patients and healthy participants. This finding is consistent with prior research emphasizing, which has emphasized the role of lipid metabolism alterations in the initiation and progression of various cancers, including neuroendocrine neoplasms (NENs)^33,34^. Lipid profile disturbances, such as an altered phosphatidylethanolamine-sphingomyelin ratio, affect lipid mobility, cell signaling, and vesicular transport, all of which are critical processes in neoplastic cell membranes^35,36^. Additionally, increased levels of polyunsaturated fatty acids (PUFA) in cell membranes reorganize lipid microdomains, altering the activity of enzymes associated with the membrane and, consequently, modulating signaling pathways^37,38^.

Dietary factors such as saturated fat intake have been linked to an increased risk of small intestinal cancer^39^. Elevated cholesterol levels have been associated with various NET types, including rectal NET^40^, gastroenteropancreatic NET (GEP NET), and pancreatic NET (PNET)^41^. These findings underscore the importance of lipid profiling, even in suspected cases of GI NET. For example, studies^42^ have demonstrated a negative correlation between HDL cholesterol levels and tumor size or progression, further supporting the role of lipid metabolism in tumor biology. The consistent performance of FTIR ratios across tumor grades contrasts sharply with CgA, whose accuracy declines in grade I NETs (sensitivity ~ 60%) and is confounded by proton pump inhibitor use^43^. While CgA remains valuable for secretory NETs, our lipid-based approach detects membrane-level changes independent of hormone secretion, potentially enabling earlier detection of non-functional tumors.

In this study, we observed changes in molecular composition and structure involving the =CH olefinic group and C–H stretching mode of HC=CH functional groups, with significant differences at 3015 cm^−1^ and 2960 cm^−1^ band. The increased absorbance ratio of I3015/I2929 in GI NET patients, as compared to healthy participants, indicates lipid peroxidation and oxidative stress within tumor cells (Fig. 5). This finding aligns with previous research showing that oxidative stress plays a pivotal role in cancer progression by inducing DNA damage and altering membrane integrity^44^.

The lipid alterations observed in the present FTIR spectra, particularly the changes in I₃₀₁₅/I₂₉₂₉ ratios, suggest severe dysregulation of lipid metabolism in GI NETs that may drive tumor progression. These spectral shifts likely reflect increased de novo lipogenesis mediated by upregulation of SREBP-1 and FASN, which have been directly implicated in NET aggressiveness and hormone secretion^45^. The elevated phospholipid turnover detected may stem from constitutive activation of the PI3K/AKT/mTOR pathway, a well-known oncogenic pathway in NETs that promotes membrane biosynthesis and cell proliferation (48). Furthermore, in the present study oxidative tumor microenvironment in NETs could induce lipid peroxidation, which may alter membrane fluidity and may contribute to the distinct FTIR signatures. These findings align with emerging lipidomic studies demonstrating that metastatic NETs undergo specific lipid reprogramming to support their invasive phenotype^46^. The FTIR data with these established mechanisms strongly suggests that lipid metabolism alterations are not merely but active participants in NET pathophysiology, thus, offering potential for biomarker development and therapeutic targeting of lipid synthesis pathways.

### Lipid-to-protein and lipid-to-lipid ratios: biomarkers for GI NETs

The spectroscopic lipid-to-lipid ratios, particularly I3015/I2929 and I3015/I1650, have emerged as potential hallmarks for distinguishing GI NET patients from healthy controls. These ratios reflect lipid peroxidation and altered protein-lipid interactions in tumor cells, which are critical indicators of oxidative stress and membrane remodeling. Our results showed higher unsaturated fatty acid content and asymmetric C–H stretching vibrations in GI NET patients. These changes, supported by robust statistical analyses, including PCA and Receiver Operating Characteristic (ROC) curve analysis, point to significant metabolic disruptions in tumor cells (Fig. 6). The observed lipid alterations, particularly elevated I3015/I2929 and I3015/I1650 ratios, suggest enhanced lipid peroxidation and oxidative stress. These findings align with the role of reactive oxygen species in modulating tumor progression and lipid membrane remodeling. Further integration of proteomic and metabolomic analyses could elucidate the mechanistic pathways underpinning these spectral changes, providing a comprehensive understanding of GI NET pathology.

ROC analysis revealed that the I3015/I2929 ratio exhibited the best sensitivity (94%) and specificity (100%) with an AUC of 0.987, while the I3015/I1650 ratio demonstrated second-best specificity and sensitivity values of 96.1% and 100%, respectively, with an AUC of 0.996 (Fig. 6c). These results suggest that lipid profiling via FTIR could be a reliable and non-invasive tool for identifying GI NETs, offering superior diagnostic accuracy compared to conventional methods.

### Clinical implications of FTIR for early detection of GI NETs

FTIR spectroscopy is a promising, cost-effective tool for GI NET screening, with practical advantages including rapid turnaround, minimal sample requirements, and compatibility with standard pathology workflows. Its potential to reduce diagnostic costs while maintaining accuracy supports future adoption in both specialized and resource-limited settings. Compared to traditional imaging techniques like Ga-68 DOTANOC PET/CT or biopsy-based histopathological assessments, FTIR offers several advantages: minimal sample requirements, radiation-free procedures, and the potential for longitudinal monitoring. This is particularly valuable for patients with rare cancers like GI NETs, where early detection is essential for improving survival rates and reducing healthcare costs.

The present study introduces a novel approach by utilizing lipid signatures in blood plasma as a diagnostic biomarker for GI NET progression. Moreover, the results open new avenues for future research into therapies targeting membrane structure, with a particular emphasis on lipid composition. Modulating the lipid environment in cancer cells may present a therapeutic strategy for disrupting tumor growth and progression.

This study provides robust preliminary evidence supporting the use of FTIR spectroscopy as a non-invasive diagnostic tool for early detection of GI NETs. By identifying significant lipid, protein, and nucleic acid alterations in blood plasma, FTIR offers unique insights into tumor biology, particularly lipid metabolism and oxidative stress in neoplastic cells. Spectroscopic lipid-to-lipid and lipid-to-protein ratios, such as I3015/I2929 and I3015/I1650, have emerged as reliable biomarkers with high diagnostic accuracy, as validated by ROC analysis (Table 3, Fig. 6c).

FTIR spectroscopy holds immense potential as an accessible, accurate, and fast screening tool that can complement existing diagnostic methods. Future studies should aim to validate these findings across larger cohorts and explore the integration of FTIR with other diagnostic technologies to enhance sensitivity and specificity further. This study also highlights the potential of lipid-centric therapeutic approaches, which could target membrane structure and signaling pathways to disrupt tumor progression.

By advancing our understanding of the molecular underpinnings of GI NETs, this research lays the foundation for future innovations in diagnostic and therapeutic strategies, ultimately improving patient outcomes in this challenging disease. While this study demonstrates the diagnostic potential of FTIR spectroscopy for GI NETs, its application in clinical settings would require addressing potential challenges such as standardization of spectral analysis, variability across devices, and the cost-effectiveness of equipment compared to other non-invasive techniques like circulating tumor DNA (ctDNA) or miRNA profiling. Future research should benchmark FTIR-based diagnostics against these methods to further validate its clinical utility. Future studies should: (1) validate these results in multicenter cohorts of 200 + patients, (2) develop AI-based spectral interpretation algorithms to standardize diagnostics, and (3) investigate exosomal lipid signatures from liquid biopsies for monitoring treatment response. Crucially, the observed lipid dysregulation reveals new therapeutic targets—particularly FASN and SREBP-1 pathways—offering avenues for drug repurposing in NET management. This work bridges the gap between spectroscopic innovation and clinical oncology, paving the way for accessible precision medicine in neuroendocrine tumor biology.

## Data Availability

The data supporting this study’s findings are available from the corresponding authors, [NR,SK, AM], upon reasonable request.

## Abbreviations

GI NET: Gastrointestinal neuroendocrine tumors
FTIR: Fourier transform infrared spectroscopy
ATR: Attenuated total reflection
